# Multipotent Nestin-Positive Stem Cells Reside in the Stroma of Human Eccrine and Apocrine Sweat Glands and Can Be Propagated Robustly *In Vitro*

**DOI:** 10.1371/journal.pone.0078365

**Published:** 2013-10-24

**Authors:** Sabine Nagel, Franziska Rohr, Caroline Weber, Janina Kier, Frank Siemers, Charli Kruse, Sandra Danner, Matthias Brandenburger, Anna Emilia Matthiessen

**Affiliations:** 1 Fraunhofer Research Institution for Marine Biotechnology, Lübeck, Germany; 2 Department of Plastic and Hand Surgery, University of Lübeck, Lübeck, Germany; ENEA, Italy

## Abstract

Human skin harbours multiple different stem cell populations. In contrast to the relatively well-characterized niches of epidermal and hair follicle stem cells, the localization and niches of stem cells in other human skin compartments are as yet insufficiently investigated. Previously, we had shown in a pilot study that human sweat gland stroma contains Nestin-positive stem cells. Isolated sweat gland stroma-derived stem cells (SGSCs) proliferated *in vitro* and expressed Nestin in 80% of the cells. In this study, we were able to determine the precise localization of Nestin-positive cells in both eccrine and apocrine sweat glands of human axillary skin. We established a reproducible isolation procedure and characterized the spontaneous, long-lasting multipotent differentiation capacity of SGSCs. Thereby, a pronounced ectodermal differentiation was observed. Moreover, the secretion of prominent cytokines demonstrated the immunological potential of SGSCs. The comparison to human adult epidermal stem cells (EpiSCs) and bone marrow stem cells (BMSCs) revealed differences in protein expression and differentiation capacity. Furthermore, we found a coexpression of the stem cell markers Nestin and Iα6 within SGSCs and human sweat gland stroma. In conclusion the initial results of the pilot study were confirmed, indicating that human sweat glands are a new source of unique stem cells with multilineage differentiation potential, high proliferation capacity and remarkable self renewal. With regard to the easy accessibility of skin tissue biopsies, an autologous application of SGSCs in clinical therapies appears promising.

## Introduction

Adult stem cells are considered to be the source for the restitution of lost cells during wound healing. Therefore, they are recognized as key players in tissue regeneration. In contrast to most other tissues, skin is an easily accessible tissue for the isolation of adult autologous stem cells. Nestin is commonly accepted as a marker protein for neural progenitor cells [1–4]. In addition, Nestin-positive cells actually meet criteria of adult stem cells, like proliferation, migration and multipotency [5–11]. The isolation and propagation of Nestin-positive stem cells was performed for tissues like skin and glandular organs [7,12–15]. Nestin-expressing cells are located in the stroma of human skin appendages like the hair follicles (connective tissue sheath and dermal papilla) as well as the sebaceous glands and most prominently in sweat glands [16,17]. *In vivo* and *in vitro* data on the regenerative potential of mammalian Nestin-positive stem cells, demonstrated various application fields for ectodermal regeneration. Thus, Nestin-positive cells derived from rodent hair follicles have already been documented to differentiate *in vitro* to neurons, glial cells, keratinocytes, and other cell types [7]. Moreover these cells can promote regeneration of peripheral nerve and spinal cord injuries upon injection to the injured nerve or spinal cord [18,19]. In addition, skin wound healing impact was also verified using rodent derived Nestin-positive stem cells [20,21]. In terms of feasibility, the use of an easily obtainable tissue with high yield of Nestin-positive cells is crucial for a successful clinical application.

Therefore, we further focused on the isolation of Nestin-positive cells from skin. Recently, we have succeeded in the isolation of Nestin-positive sweat gland stroma-derived stem cells (SGSCs), which showed high proliferation activity and substantial differentiation plasticity [16]. In addition, we have shown in preclinical study, that transplantation of matrices treated with human SGSCs into full thickness skin defects in mice significantly improved vascularization [22]. 

Furthermore, human eccrine sweat gland cells revealed similar wound healing ability compared to keratinocytes. Biedermann et al. demonstrated the capability of human eccrine sweat gland cells to form a stratified interfollicular epidermis substitute on collagen hydrogels both *in vitro* and *in vivo* [23]. A recently published paper analyzed the wound healing capacity of murine eccrine sweat glands *in vivo* and evidenced the existence of different multi- and unipotent cells in sweat glands [25]. Furthermore, they could demonstrate that ductal epithelial cells are involved in epidermal regeneration processes. Rittie et al. verified an equal outcome for human eccrine sweat glands *in vivo* [24]. 

Even though the rising interest in Nestin-positive cells as well as in sweat glands in relation to wound healing, many questions still remain. Thus, the exact localization and origin of the Nestin-positive cells in human eccrine and apocrine sweat glands is unclear. In addition, it is unknown if Nestin-positive cells can reproducibly be isolated from human sweat glands and if parameters exist that allow propagation conserving stem cell properties. Finally the potential of sweat gland derived Nestin-positive cells for skin wound healing needs to be analyzed. Therefore, investigations concerning those issues have been carried out by us and are presented here. 

## Results

### Localization of Nestin-positive cells in eccrine and apocrine human sweat glands

**Figure 1:**
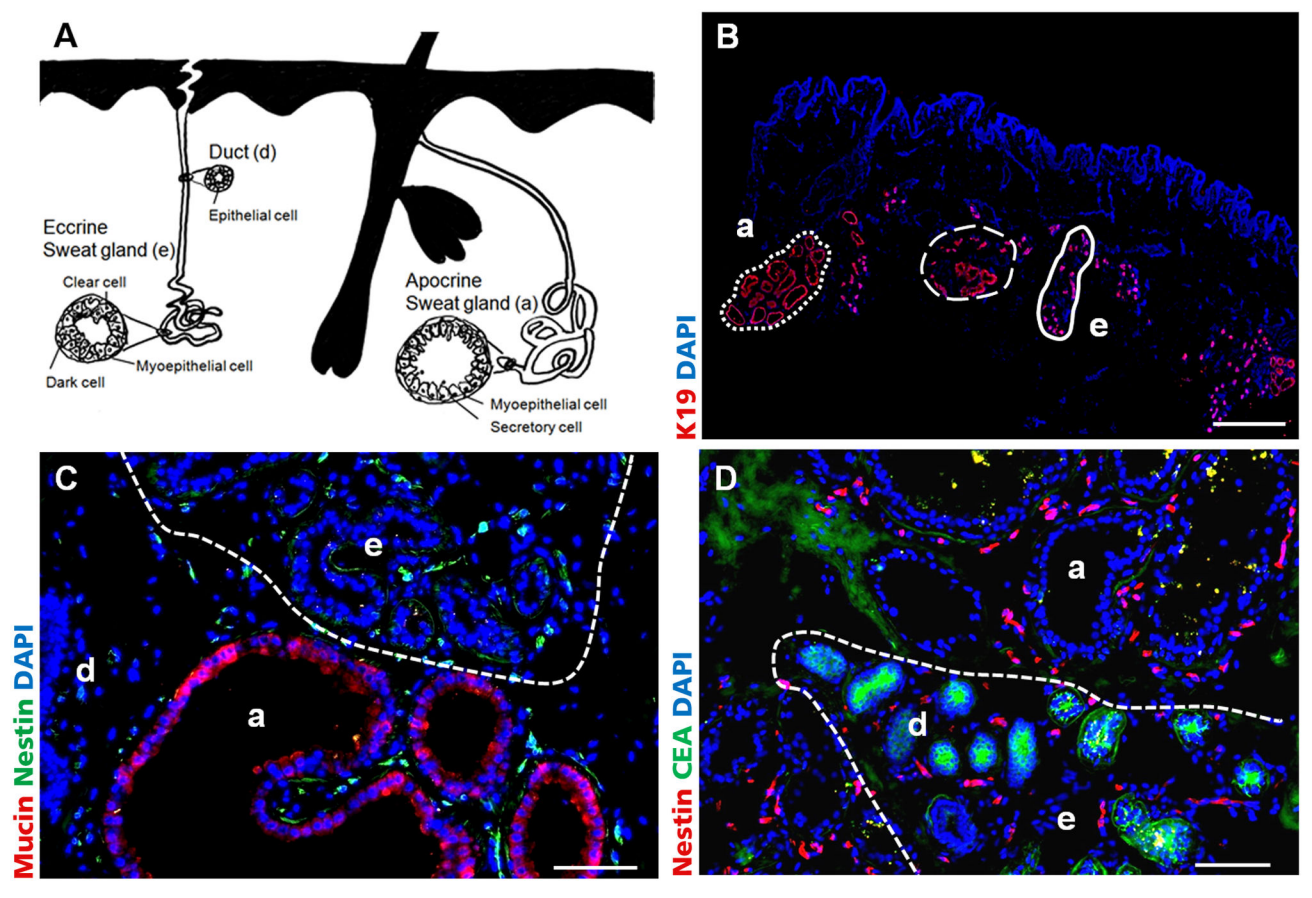
Localization of Nestin-positive cells in human sweat glands of axillary skin. A) Schema of the human skin appendages, with special focus on localization and structure of eccrine (e) and apocrine (a) sweat glands. B) K19 (red) IF staining of apocrine (a, dashed line) and eccrine (e, full line) sweat glands in human axillary skin. Eccrine and apocrine glands are in immediate vicinity, sometimes they were entwined around each other (halted line). Nuclei were stained with DAPI. Scale bar 1000 μm C) IF staining of Mucin. Secretory cells in apocrine (a) sweat glands are positive for Mucin (red), whereas eccrine (e) sweat glands are negative (halted line). D) IF staining of carcino-embryonic antigen (CEA). CEA (green) was expressed in ducts (d) and the secretory part of eccrine (e) sweat glands (halted line). Nestin (C green, D red) positive cells could be found in the stroma of apocrine and eccrine sweat glands. Scale bars 100 μm.

The schematic illustration in Figure 1A shows the most important human skin appendages, focusing on localization and structure of eccrine and apocrine sweat glands. A histological overview of human sweat glands in axillary skin is shown in Figure 1B. In adult, healthy human skin K19 expression is restricted to sweat glands and single cells in hair follicles. It was easy to distinguish between eccrine (e, full line) and apocrine (a, dashed line) sweat glands via their size. The lumen of apocrine glands is 10 times larger in diameter than the eccrine gland lumen. The predominant localization area of eccrine glands is the dermis, whereas the apocrine glands are present deeper in the dermis and subcutis. Eccrine and apocrine glands are in immediate vicinity, sometimes they were entwined around each other (Figure 1B, halted line). Axillary skin tissue sections have been analyzed for the localization of Nestin-expressing cells. Discrimination of eccrine and apocrine sweat glands was additionally achieved by immunofluorescence (IF) staining of carcinoembryonic antigen (CEA) and Mucin (Figure 1C, D). CEA was expressed by ductal cells of both sweat gland types and in addition by secretory cells of eccrine sweat glands, whereas Mucin was expressed by secretory cells of apocrine sweat glands only. In both sweat gland types Nestin-positive cells could be detected in the stroma between secretory coiled glands and ducts (Figure 1C, D). 

### Reproducibility of isolation and propagation of Nestin-positive cells

**Figure 2:**
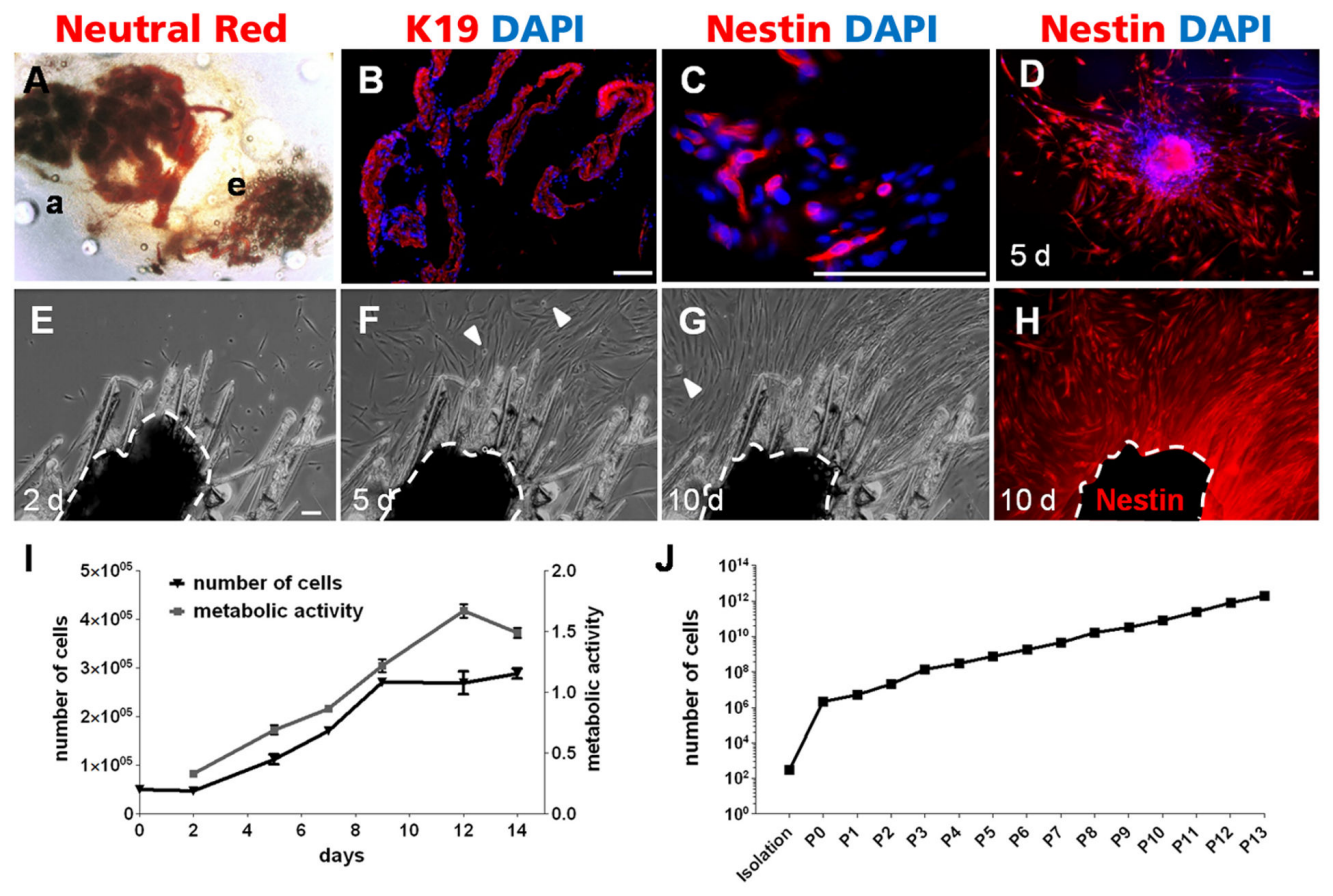
Isolation and growth properties of Nestin-positive cells from human sweat glands. A) Apocrine (a) and eccrine (e) sweat gland after enzymatic and mechanical isolation stained with neutral red. B) Isolated tissue was exclusively K19 positive, demonstrating successful isolation of pure sweat glands. C) Nestin-positive cells were still detectable in the stroma of the isolated sweat glands. D) After 5 days of cultivation nearly every outgrowing cell was Nestin-positive. Nuclei were stained with DAPI. E–H) Recordings of the time-lapse movie following 10 days of cell outgrowth from a single human sweat gland (halted line) to a confluent cell layer. E) 2 days after isolation, the first cells migrated out of the sweat gland. F) 5 days after isolation, cells around the sweat gland propagated through migration and division (white arrow heads: mitotic cells). G) A confluent cell layer was reached after 10 days. H) At this confluence state IF staining revealed Nestin expression in nearly every outgrowing cell. The auto fluorescence of the sweat gland was covered in black. Scale bars 100 μm. I) Growth characteristics of SGSCs during *in* *vitro* propagation over 14 days by determining cell number and metabolic activity (MTT turnover) (n=3, mean±SEM). J) Long-term proliferation ability within 13 passages of SGSCs propagation was evaluated. The cell number increased consistently over the subsequent passages and even after the 12th passage there was no proliferation slowdown or replicative senescence detectable (n=3, mean±SEM).

After mechanical and enzymatic treatment of axillary skin, eccrine and apocrine glands could clearly be identified with a neutral red staining and discriminated via their morphology and size (Figure 2A). A high purity of sweat gland tissue, free of adjacent skin remnants could be achieved with this method. This could be verified demonstrating exclusive K19 expression within this tissue (Figure 2B). Furthermore, Nestin-positive cells were still found in the stroma and directly present at the boundaries of the gland (Figure 2C). After immobilization of isolated sweat glands at the bottom of a coated culture dish, cells migrated out of the sweat gland stroma within 5 days. Almost all outgrowing cells were Nestin-positive (Figure 2D). 

The outgrowth of cells from the isolated sweat glands was documented by time lapse analysis and resulted in a film sequence from day 2 until day 10 (Figure 2E-H). Two days after isolation, the first cells migrated out of the sweat gland (Figure 2E). Within 5 days the cells around the sweat gland propagated through migration and division (see white arrow heads=mitotic cells) (Figure 2F). At day 10, a confluent cell layer around the sweat gland was visible (Figure 2G). At this confluence state an IF staining of the specimens revealed Nestin expression in nearly every outgrowing cell (Figure 2H). Note that the sweat gland size decreased from day 2 until day 10 (halted line).

To analyze the growth characteristics of SGSCs during *in vitro* propagation the cell number was determined over 14 days at 6 different time points (Figure 2I). The population doubling time during the exponential growth phase between day 2 and day 9 was 2.8 days. Afterwards, the cells reached a confluent state and proliferation slowed down. Consistent with the increasing cell number the estimated overall metabolic activity (MTT turnover) increased continuously over time and decreased after the confluent state was reached. In further analyses the long-term proliferation ability was evaluated over several passages (Figure 2J). The cell number increased consistently from isolation to passage 13 and no proliferation slowdown or replicative senescence was observable.

### After *in vitro* assimilation, SGSCs maintained their spontaneous, multipotent differentiation capacity and secreted cytokines during propagation

**Figure 3:**
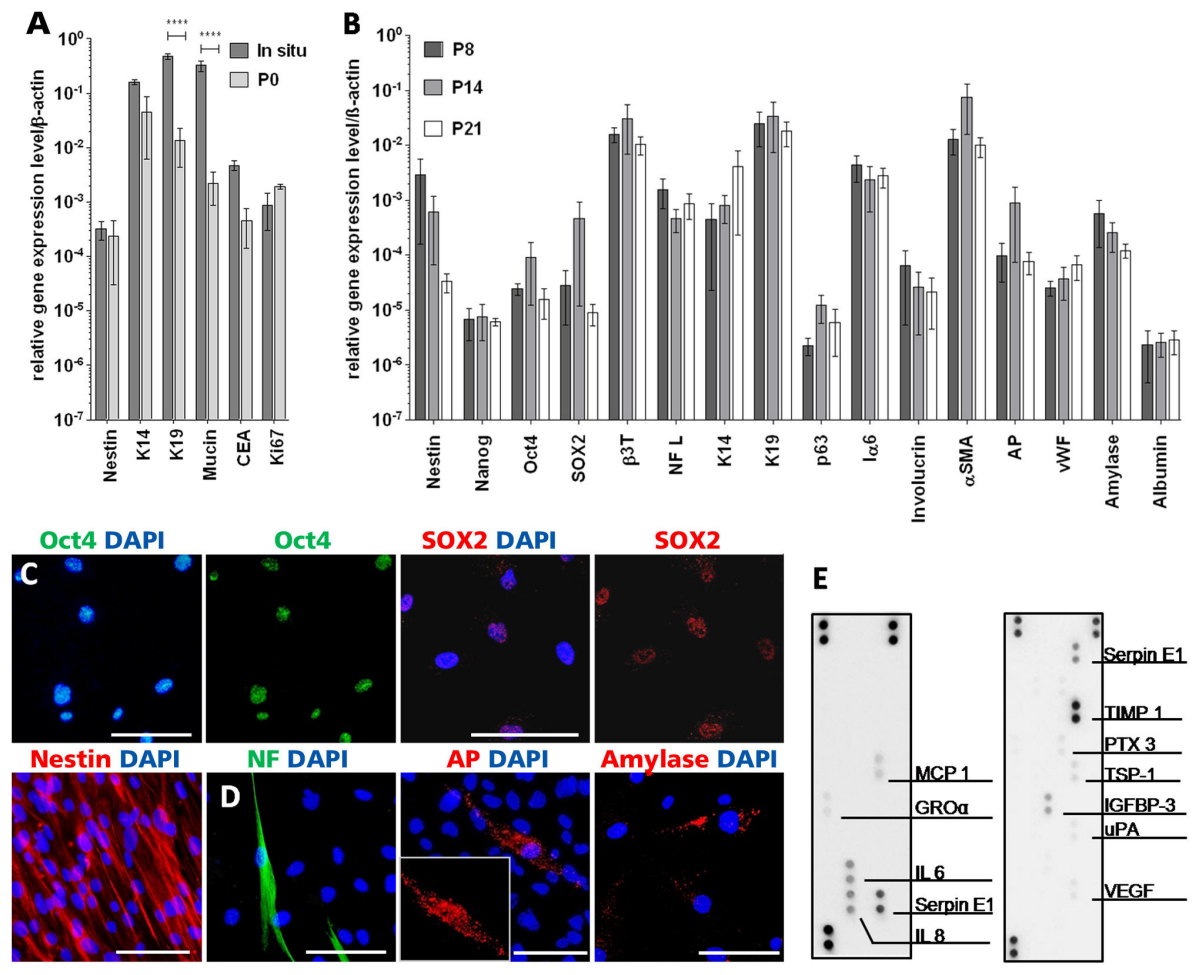
*In* *vitro* characterization of SGSCs. A) Gene expression profile analysis via qPCR from sweat glands directly after isolation compared to outgrowing cells. Distinct changes in gene expression could be detected (n=3, mean±SEM). Whereas Nestin was constantly expressed, expression of sweat gland related genes (K14, K19, Mucin, CEA) decreased during *in* *vitro* cell propagation. In contrast transcripts indicating proliferation (Ki67) increased. **** p<0,0001 B) qPCR was performed to determine the expression of transcripts corroborating a multipotent differentiation capability and to evaluate expression variations between passages (P8, P14, P21) and donors (n=3, mean±SEM). There were no significant variations in gene expression levels. C) Expression of stem cell-related proteins Oct4, SOX2 and Nestin in SGSCs. D) IF analysis of proteins specific for cells of ectodermal (NF), mesodermal (AP) and endodermal (Amylase) origin. Nuclei were stained with DAPI. Scale bars 100 μm. E) Analysis of cytokine secretion via membrane-based array system of SGSCs grown on cell culture plastic. Via cytokine array (first panel) and angiogenesis array (second panel), factors involved in vascularization, immune regulation and tissue remodeling could be detected.

The analysis of the expression profile of SGSCs via quantitative PCR (qPCR) was used to determine changes from *in situ* to *in vitro* cultivation. Therefore, mRNA level from freshly isolated sweat glands and outgrowing cells were compared using 3 different donors (Figure 3A). Whereas Nestin was constantly expressed, the sweat gland related transcripts K19 and Mucin decreased significantly (p<0,001 ****). K14 and CEA, ,) declined likewise, but not significantly. In contrast Ki67 expression increased (n.s.) .

Additional expression analyses were performed to determine the expression of transcripts corroborating a multipotent differentiation capability (Figure 3B). Therefore, expression profile was preserved in early and late passages (P8, P14 up to P21) using 3 different donors. It could be shown that there were no significant variations in the expression levels over different passages. The stem cell properties of SGSCs were underlined by the detection of transcripts related to multipotency like Nestin, Nanog, Oct4 and SOX2. The expression of various genes, which are involved in the differentiation towards cell types of the 3 embryonic germ layers (ectodermal: β3T, NF L, mesodermal: αSMA, AP, vWF and endodermal: Amylase, Albumin) was verifiable. Because appendages originate from the ectodermal part of the skin, we focused on the analysis of mRNAs of various skin and sweat gland associated keratins (K14, K19), transcripts for cells of the basal layer (p63, Iα6) and transcripts related to skin differentiation (involucrin). All of these transcripts were determined in SGSCs. 

The multipotent differentiation potential of SGSCs was also shown on protein level. IF staining revealed expression of the stem cell markers Oct4, SOX2 and Nestin (Figure 3C). All markers related to cell populations with multilineage differentiation potential [5,26,27]. Their potential to differentiate spontaneously into cell types of the 3 embryonic germ layers *in vitro* was evaluated by IF staining for proteins specifically expressed in ectodermal (NF), mesodermal (AP) and endodermal (Amylase) cells (Figure 3D). 

With regard to a clinical application of SGSCs, cytokine secretion was analyzed by a specific proteome profiler array. Cytokines like monocyte chemotactic protein 1 (MCP 1), growth related oncogene alpha (GROα), interleukin 6 (IL 6) and 8 (IL 8) as well as serine proteinase inhibitor (Serpin E1) were detected (Figure 3E, left panel). The specific proteome profiler array evaluating angiogenesis related cytokines revealed the secretion of Serpin E1, tissue inhibitor of metalloproteinases 1 (TIMP 1), pentraxin 3 (PTX 3), thrombospondin-1 (TSP-1), insulin-like growth factor binding protein 3 (IGFBP-3), urokinase plasminogen activator (uPA) and vascular endothelial growth factor (VEGF) (Figure 3E, right panel). In the related negative control only the positive spots were visible (data not shown). 

### SGSCs coexpressed Nestin and Integrin αlpha 6

**Figure 4:**
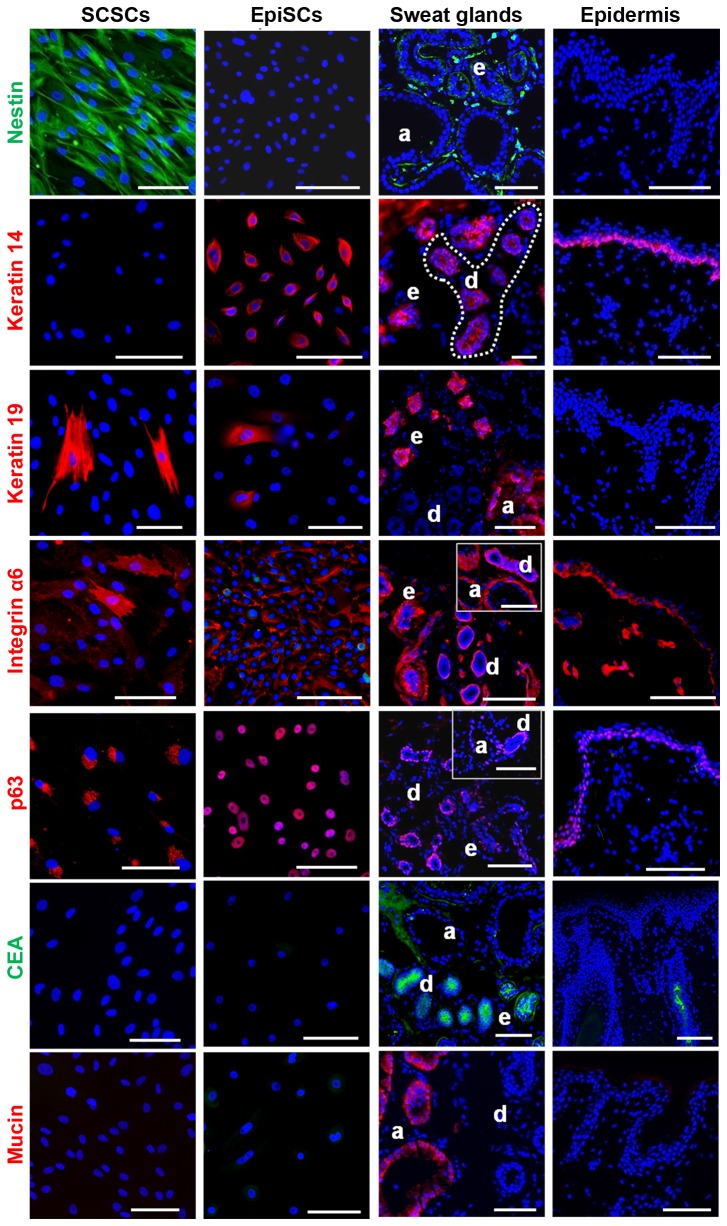
Localization of skin and sweat gland-related proteins *in* *vitro* and *in* *situ* by IF staining. Direct expression comparison of SGSCs (first panel), EpiSCs (second panel), sweat glands (third panel) and human axillary skin (fourth panel). This overview confirmed that the expression profile of SGSCs is different from EpiSCs. Nuclei were stained with DAPI. Scale bars 100 μm. Apocrine (a) and eccrine (e) sweat gland, duct (d).

The protein expression of SGSCs was compared to that of EpiSCs and to the *in situ* expression in sweat glands and epidermis (Figure 4). Nestin, which was detected in SGSCs and sweat glands, was neither expressed by EpiSCs nor in epidermis. In contrast to EpiSCs, SGSCs did not express K14, a protein found in stratified skin layer cells. However, K14 was detectable in the duct and the myoepithelial cells of the sweat glands as well as in basal cells of the skin. Nevertheless, there were also similarities in protein expression between SGSCs and EpiSCs. It could be shown that K19, which was expressed by EpiSCs and SGSCs, was also present in sweat glands *in situ*. Additionally, integrin alpha 6 (Iα6) was detected in all samples. It was expressed homogenously on the total surface of the SGSCs, whereas the intensity varied between cells. In contrast, EpiSCs expressed Iα6 predominantly at the cell-matrix contacts. In sweat glands and skin, Iα6 expression was not restricted to the basal cells, since positive cells existed in the stroma as well. Furthermore, the nuclear epidermal stem cell marker p63 was detected in SGSCs (only in the endoplasmatic reticulum) and EpiSCs. In sweat glands p63 was expressed by ductal basal cells and the myoepithelial cells of the secretory parts. In skin p63 was only detected in basal cells. In contrast, the sweat gland associated proteins Mucin and CEA were neither expressed by SGSCs nor EpiSCs *in vitro.*

**Figure 5:**
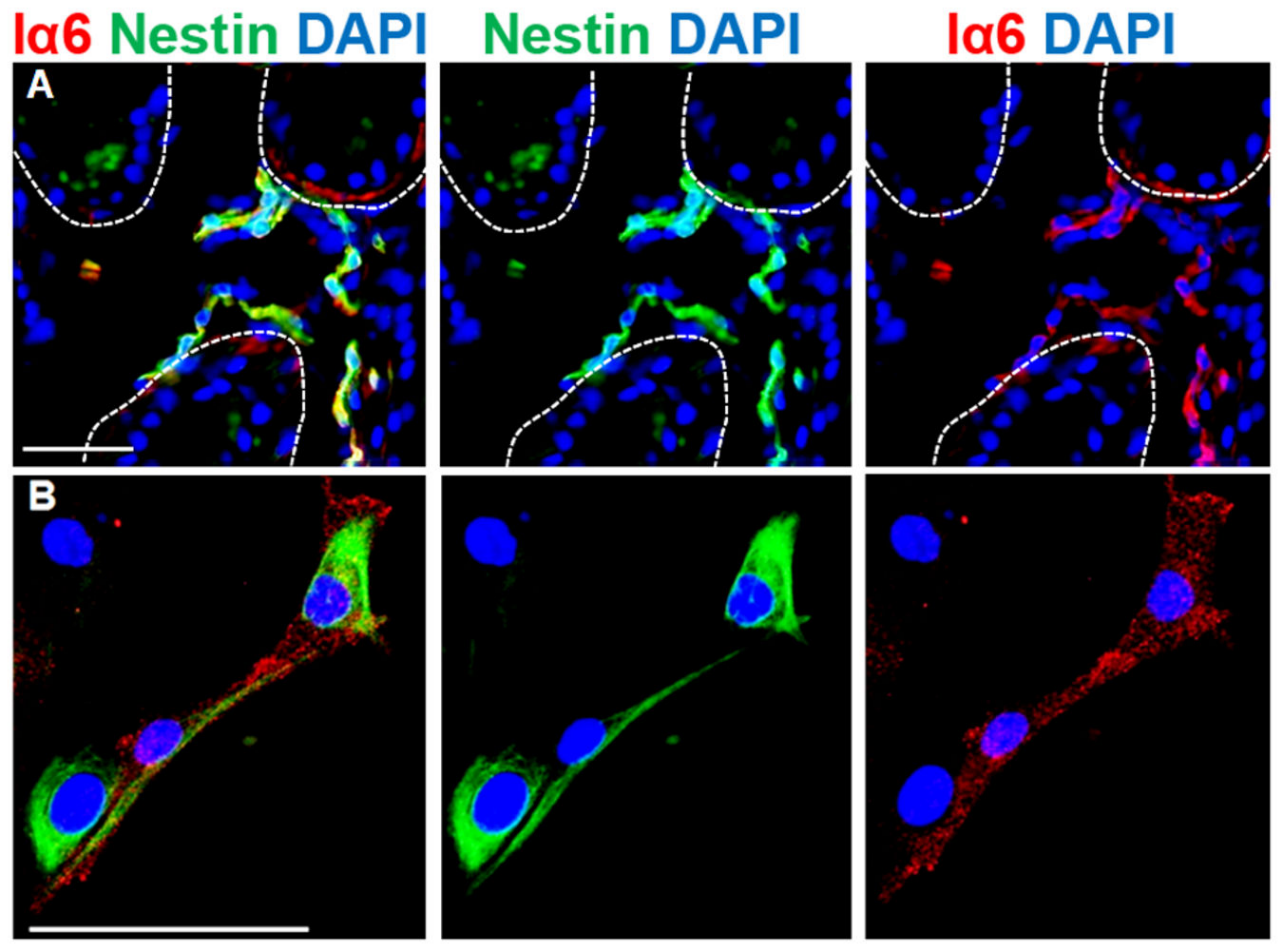
Localization of Nestin and Iα6 double positive cells *in* *situ* and *in* *vitro* by IF staining. A) Double positive cells could be verified in the stroma of sweat glands (halted line). B) Double positive cells could also be detected in SGSCs *in* *vitro*. Nuclei were stained with DAPI. Scale bars 100 μm.

Overall, SGSCs could clearly be distinguished from EpiSCs, since they expressed markers for basal cells but did not express proteins of stratified skin layer cells. To identify the potential origin of SGSCs *in situ*, we performed an additional double IF staining of Iα6 and Nestin in axillary skin (Figure 5). It could be shown that cells of the sweat gland stroma predominantly but not exclusively coexpressed Nestin and Iα6 (Figure 5A), which could also be confirmed *in vitro* (Figure 5B).

### Mesodermal differentiation via soluble factors

**Figure 6:**
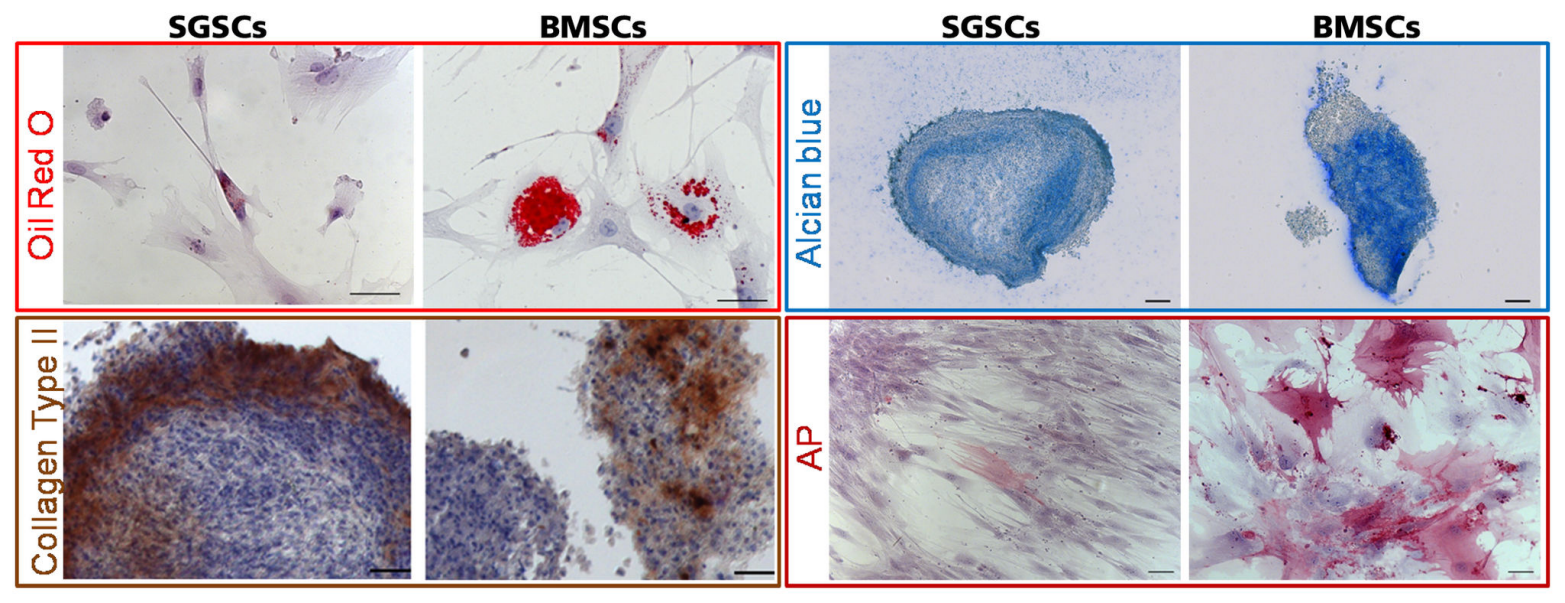
Induced differentiation of SGSCs via soluble factors in comparison to BMSCs over 21 days. For confirmation of adipogenic differentiation fat droplets were documented with Oil Red O staining. Proper differentiation was shown for BMSCs by the existence of several cells containing red stained lipid droplets. In stimulated SGSCs there were only single cells with small lipid droplets. Nuclei were stained with haematoxilin. Scale bars 100 μm Alcian blue staining was used to detect chondrogenic differentiation of micromass bodies. SGSCs and BMSCs showed a distinct staining. Scale bars 200 μm Staining for Collagen type II was positive in both, but stronger expressed by SGSCs in comparison to BMSCs. Nuclei were stained with haematoxylin. Scale bars 100 μm Proper differentiation into the osteogenic lineage could be proven for BMSCs by the existence of several cells expressing alkaline phosphatase (AP). For SGSCs only few cells were stained. Nuclei were stained with haematoxylin. Scale bars 100 μm.

Targeted differentiation towards adipogenic, chondrogenic and osteogenic cells was carried out using SGSCs in comparison to BMSCs (Figure 6). Adipogenic differentiation was proven for stimulated BMSCs by the existence of several cells containing red stained lipid droplets were detected. In stimulated SGSCs only single cells contained small lipid droplets. For verification of chondrogenic differentiation micromass bodies (MMBs) were prepared. SGSCs generated compact MMBs, while BMSCs only formed irregular shaped MMBs with spongy specimens. However, a distinct alcian blue staining was detectable for both, SGSCs and BMSCs. Furthermore, staining for Collagen type II was also positive in both, but stronger expressed by SGSCs in comparison to BMSCs. Proper differentiation into the osteogenic lineage could be proven for BMSCs by the existence of several cells expressing alkaline phosphatase (AP). For SGSCs only few cells were stained. Except of a slight alcian blue staining at the edges of the very spongy MMBs of the controls, no positive cells could be detected within the corresponding negative controls (Figure S1).

## Discussion

First, we proved the existence of Nestin-positive cells in the sweat gland stroma of human eccrine and apocrine sweat glands within the secretory as well as the ductal parts. With regard to a possible clinical application we established a reproducible isolation and *in vitro* propagation of SGSCs without changing their spontaneous, multipotent differentiation capacity significantly. Additionally, we verified the immunological potential of SGSCs to promote skin wound healing. It was also possible to distinguish SGSCs from EpiSCs and BMSCs by their specific expression profile and their particular differentiation capacity. Furthermore, we were able to determine a coexpression of Nestin and Iα6 in SGSCs as well as in human sweat gland stroma.

In this study we succeeded in precisely locating the origin of the Nestin-expressing cells within human axillary skin. Besides hair follicles and sebaceous glands Nestin-positive cells could be detected in the stroma of apocrine and eccrine sweat glands, associated with secretory and ductal compartments. Since Nestin is an intracellular intermediate filament protein, there was no possibility to sort vital Nestin-positive stem cells via fluorescence activated cell sorting using specific surface antibodies as it is common for mesenchymal stem cells from bone marrow or fat tissue. For later therapeutical application, a high-yield isolation procedure of Nestin-positive cells is crucial. Even though preparations from full skin provide a moderate yield of about 30% of Nestin-expressing cells [12,28], no one else has isolated Nestin-positive stem cell populations from sweat glands so far. Therefore, the identification of the origin of Nestin-positive cells is crucial for further optimization of the isolation procedure. By observing the outgrowth of the cells using time lapse microscopy in combination with an IF staining of Nestin at the endpoint, we tried to examine the origin of Nestin-positive cells. We observed a decrease in sweat gland size, while cells migrating out of it. Furthermore, mitotic activity among the outgrowing cells became visible so that the *in vitro* cell population is a result of migration and proliferation of sweat gland cells. Since the Nestin-expressing cells have not been tagged directly *in situ*, the tracking of these cells was not possible so far. However it was obvious, that Nestin-positive cells were located in the proximity of sweat glands, giving a hint that these cells could have contributed to the resulting cell population. Amoh et al. showed that it is possible to detect GFP-Nestin murine hair follicle cells migrating out of the hair follicle, but it is still unclear whether only those Nestin-positive cells contributed to the resulting cell population or if other cell types also started to express Nestin *in vitro* [19]. 

We hypothesize a parallelism of stem cell function in different compartments for all Nestin-positive cells in the stroma of skin appendages. A possible explanation for that theory is that all appendages arise from the epidermal ridge during embryogenesis [29–32]. The epithelial-mesenchymal cross-talk within all these appendages might be regulated by Nestin-positive cells in the stroma. This interaction is important not only during embryogenesis but also during hair cycling, activation during adolescence (apocrine sweat glands) and regeneration. Anyhow, skin appendages go through many morphological and/or physiological changes during their life time. In addition, the characteristic property of releasing cell components or whole cells during secretion is common across mammary, sebaceous and apocrine sweat glands. Due to this secreting activity the homeostasis of exocrine glands necessitates a population of progenitor cells, which can constantly regenerate the lost specialized cells. Overall, the existence of Nestin-positive was demonstrated and some have been assigned also to the stroma [17,33,34]. It could be assumed that Nestin-positive stem cells are involved in all these mechanisms. The comparison of gene expression between freshly isolated sweat glands and outgrowing cells showed partly significant changes. We observed a decrease in expression of all sweat gland-related transcripts, such as K14, K19, CEA and Mucin. Probably these differentiated cells got lost during *in vitro* culture. Another explanation for this change in expression patterns could be a switch in cell identity and partial reprogramming, so that functional differentiated sweat gland cells stop to synthesize sweat gland-related transcripts and start to express stem cell related genes. A similar process has been observed from Rapoport et al. for *in vitro* culture of exocrine pancreatic cells that stop to produce amylase and start to express Nestin instead [35]. But Nestin gene expression of freshly isolated sweat glands and outgrowing cells was nearly constant, although nearly every outgrowing cell expressed Nestin on protein level. A possible explanation could be the late time point of cell harvesting, where the cell population was highly confluent. Because Nestin is mainly expressed in migrating and mitotic cells, transcription rate of Nestin at this confluence could already be down regulated, whereas the protein is highly expressed. 

After the outgrowth of SGSCs we obtained a constantly proliferating cell population with long-term growth capabilities. This could be explained by the increased expression of the proliferation-associated transcript of Ki67. Because of the generation of clinically necessary cell amounts (more than 10 million cells in 30 days, data not shown) in relatively short time this cell population holds promise for human cell-based therapies. Thereby no changes in ploidity could be observed within analysed SGSCs during propagation (Figure S2). However, we currently establish precise quality parameters for monitoring SGSCs during the whole process of *in vitro* cultivation. 

In addition to the proliferation capabilities of SGSCs their stem cell properties were underlined by the detection of Oct4 and SOX2 which related to multipotency and plasticity [36–38]. However the expression pattern seemed to be diverse compared to embryonic stem cells. The protein expression of Oct4 and SOX2 in SGSCs was relatively weak and was localized as punctual pattern in the nucleus. This finding was observed in other adult stem cell populations as well [39,40]. However, gene expression of all measured transcripts remained nearly constant without any significant variation between passage 8 and 21. Nevertheless, further investigation is needed to verify this trend. The expression of stem cell related proteins and proteins expressed by cell types of the 3 embryonic germ layers could also be proven. Taken together the analysis resulted in clearly defined marker genes for this cell population. 

Beside their multipotent differentiation capacity, SGSCs were able to secrete cytokines which play a role in immune regulation (IL 6, IL 8, MCP 1, GROα, PXT 3), vascularization (VEGF, TSP-1) and tissue remodeling (Serpin E1, uPA, TIMP 1) [41–45], demonstrating an immunological potential of SGSCs. Paracrine signaling during wound healing is a highly complex and regulated process with cross-talk of resident cell types and immune cells. By secretion of cytokines transplanted SGSCs could modulate immune reactions, tissue remodeling and processes in angiogenesis. This substantial wound healing-promoting potential of Nestin-positive SGSCs was demonstrated *in vivo* before [22]. 

Since Biedermann et al. established the isolation of keratinocyte progenitor cells from sweat glands [23], it was also an imperative necessity to discriminate SGSCs from special keratinocyte subpopulations. For precise distinctions we compared protein expression patterns of SGSCs and EpiSCs *in vitro* with sweat glands and skin *in situ*. The significant difference between SGSCs and EpiSCs was the expression of the key marker Nestin, which was neither expressed by EpiSCs nor epidermis but by SGSCs and in the sweat gland stroma. Especially striking is the absence of K14 in SGSCs in comparison to EpiSCs. This filament protein is known to be expressed in stratified basal epithelia [46], in the outer root sheath of the hair follicle [47] and in sweat glands by the ductal and myoepithelial cells [48]. K19, which is expressed in basal cells of human epidermis in an age dependent manner and is lost in adultness [49], was detectable in SGSCs and EpiSCs. There are several reports stating that K19-expression is correlated with self renewal [50–52]. In hair follicles K19 is expressed in the bulge region and the outer root sheath [53]. Integrin alpha 6 (Iα6) could be detected in SGSCs, in EpiSCs as well as in sweat glands and basal cells of the epidermis. Additionally Iα6 is expressed in hair follicles [53]. In a double IF staining it could be proven that Nestin-positive cells in the sweat gland stroma also expressed Iα6. Furthermore, double positive cells could also be verified *in vitro*. Iα6 plays a role during migration [54], cell survival [55] and signal transduction [56]. Interestingly, a role for this cell surface protein in regulating differentiation and maintaining pluripotency is suggested, as it regulates the stem cell markers Oct4 and SOX2 [57]. Iα6 is in turn regulated by p63 [58], which is a transcription factor and a lineage-specific factor of the proliferative capacity in stem cells in human epidermis and hair follicles [59,60]. With this finding, it might be possible to enrich Nestin-positive cells by Iα6 as a surface marker. In sweat glands p63 was expressed by ductal and myoepithelial cells. SGSCs expressed p63 only in the endoplasmatic reticulum, which could represent the ability to synthesize p63 protein, but it may not be functionally expressed in the nucleus. EpiSCs demonstrated proper p63 expression in the nucleus of nearly every cell. 

Taken together these results imply that the SGSCs special origin in the stroma of sweat glands endows them with particular characteristics such as Nestin and Iα6 expression and distinguishes them from classical stem cells of epidermal origin. With Iα6 as a surface marker, it might be possible to enrich Nestin-positive cells. 

Other groups have already shown the presence of stem cell populations in the eccrine sweat glands, with a phenotype more linked to keratinocyte stem cells [23]. Based on different isolation and cultivation protocols, it is likely that Biedermann et al. have different cell populations compared to SGSCs. Therefore, sweat glands are of major importance because they harbor not only Nestin-positive SGSCs but also keratinocyte progenitor cells. 

Since SGSCs could be discriminated from EpiSCs, another feasible origin of these cells is the stroma, which is also the niche of the Nestin-positive cells *in situ*. A crucial property of stem cells of the mesenchymal stroma is their mesodermal differentiation potential. Determination of the differentiation capability of the SGSCs in comparison to BMSCs gave further insights into the origin of these cells. For BMSCs it was repeatedly shown that they can differentiate towards adipocytes, chondrocytes and bone cells by treating them with specific differentiation media [61,62]. Obviously, SGSCs have not the common mesodermal differentiation potential of BMSCs towards mesodermal lineages. Thus, SGSCs have a lower potential to differentiate into fat and bone under the applied conditions, but they have the capacity to differentiate into cartilage. 

Not only differentiation capabilities differs SGSCs from BMSCs but also the expression of the key marker Nestin. Nestin was expressed in only <5% of the BMSCs (Figure S3A). Furthermore, we analyzed dermal fibroblasts, which expressed Nestin in <1% of the cells. Vimentin, a protein which is expressed by mesenchymal cells, is also positive in SGSCs (Figure S3B). In addition, it can be found in MSCs but also in endothelial cells, fibroblasts and neural cells especially during development and regeneration [63–65]. Vimentin is an intermediate filament and often coexpressed with Nestin. Furthermore, there is a functional interaction between Vimentin and Nestin building up heterofilaments mainly in dynamic cell populations with high migration and proliferation capacity [11,66–68]. Taken together these results imply that there are similarities of SGSCs and EpiSCs, BMSCs and dermal fibroblasts but the extraordinary Nestin expression distinguishes SGSCs from all these cell populations.

Other Nestin-positive cell populations existing in skin are neural crest cells [69–71], endothelial cells [72,73] and periendothelial cells [64]. Current investigations analyze similarities between these Nestin-positive cells with SGSCs. First results demonstrated that SGSCs expressed special markers of neural crest cells (NGFR, SOX9) and endothelial cells (PECAM) (Figure S4). But further investigation is needed to confirm these data. In conclusion, we demonstrated a novel Nestin-positive stem cell population from human sweat glands having long-time propagation ability without changing their spontaneous multipotent differentiation capacity extensively. Additionally, we verified the immunological potential of SGSCs, which demonstrated their undisputable regenerative potential and promising clinical application to promote skin wound healing. Furthermore, we verified a novel coexpression of Iα6 and Nestin in SGSCs and in sweat gland stroma. The impact of this coexpression will be the subject-matter in continuing studies.

## Materials and Methods

### Ethics Statement

All experiments were performed according to Helsinki guidelines, in compliance with national regulations for the experimental use of human material. Utilization of human biopsies for research purposes was approved by the ethics committee of the University of Lübeck (reference number: 10–058). All patients gave written informed consent. 

### Isolation of Nestin-positive cells from human eccrine and apocrine sweat glands

A biopsy of human axillary skin was obtained from male and female donors aged between 23 and 54 years. Due to continuous improvements of the isolation protocol, the first population was isolated with a slightly modified protocol [16], but both isolation procedures resulted in connective tissue-free sweat glands. In order to isolate sweat glands with the improved protocol, an axillary skin biopsy (about 1x2 cm^2^) was cut into pieces and digested in dispase II (2 U/ml, Roche, Germany) overnight at 4°C. The following day the epidermis was removed and discarded. The dermis was shredded with scissors in digestion medium containing 0.2 mg/ml collagenase (NB8; Serva, Uetersen, Germany). Next, dermis pieces were incubated at 37°C under constant shaking (150 cycles/min) for 3 h. Afterwards it was proceeded as described in Petschnik et al. [16]. The analyses were performed with SGSCs of 6 donors to consider patients variability.

### Isolation of human epidermal stem cells

Epidermal stem cells (EpiSCs) were generated according to the fast adhering method from different regions of adult human skin [74]. Split-thickness skin grafts were produced (300 µm) by using the Dermatom D42 (Humeca, Enschede, Netherlands), reduced in 6 mm punches and incubated in dipase II (2 U/ml, Roche, Germany) for 24 h at 4°C. Afterwards epidermis and dermis were separated with forceps. Next, the epidermis was incubated with 2.5% trypsin for 5 min at 37°C and further dissociated by up and down pipetting for additional 5 min. After centrifugation, epidermal cells were resuspended in EpiLife, supplemented with 0.6% antibiotic-antimycotic (100x) and EpiLife Defined Growth Supplement (all Gibco, Darmstadt, Germany). Epidermal cells were seeded with a density of 3.5*10^4^ cells/cm^2^ on collagen type-IV coated culture dishes (BD Biosciences, Heidelberg, Germany) for 7 min at 37°C and 5% CO_2_. Supernatant was discarded and cultivation of the adherent cells was performed in EpiLife. The medium was changed every 3–4 days.

Human mesenchymal stem cells of the bone marrow (BMSCs) were purchased from PromoCell (Heidelberg, Germany). Cells were handled according to manufactures instructions. Dermal fibroblasts were also purchased from PromoCell (Heidelberg, Germany). 

### Propagation of Stem Cells

Propagation of SGSCs was performed according to Petschnik et al. [16]. In this study, cells from the isolation until passage 21 were analyzed. Dermal fibroblasts were handled like SGSCs.

EpiSCs: Cell propagation was performed in EpiLife. After reaching 80-90% confluence, cells were subcultured by treatment with 0.1% trypsin (PAA Laboratories, Austria) and reseeded in EpiLife. Medium was changed every 3-4 days. In this study, cells from passage 2-4 were analyzed.

BMSCs: Cell propagation was performed in mesenchymal stem cell growth medium [MSC-GM (PromoCell, Heidelberg, Germany), penicillin 1 U/ml and streptomycin 10 mg/ml (both PAA Laboratories, Linz, Austria)]. After reaching confluence, cells were subcultured by treatment with 0.1% trypsin (PAA Laboratories, Austria) and reseeded in MSC-GM. Medium was changed every 3-4 days. In this study, cells from passage 12-16 were analyzed.

### Analysis of SGSC growth characteristics

To analyze the growth characteristics of SGSCs their proliferation and metabolic activity was estimated during *ex vivo* expansion. SGSCs were seeded into 6 well plates in triplicates (5*10^4^ per well). Growth medium supplemented with 10% FCS [DMEM-10: Dulbecco’s modified Eagle’s medium (Gibco, Karlsruhe, Germany), penicillin 1 U/ml and streptomycin 10 mg/ml (all PAA Laboratories, Linz, Austria)] was replaced every 3-4 days. Every 2-3 days within 14 days, SGSCs were analyzed in two different ways. First, SGSCs were trypsinized and counted using an automatic cell counter (NC-100, Chemometec, Allerød, Denmark). Second, SGSCs were incubated for 1 h in DMEM-10 containing 0,5 mg/ml of 3-(4, 5-dimethyl-2-thiazolyl)-2, 5-diphenyl-2H-tetrazolium bromide (MTT, Roth) at 37°C and 5% CO_2_. Next, medium was removed, cells were washed with phosphate buffered saline (PBS) and incubated with 300 μl of dimethyl sulfoxide per well (DMSO, Sigma-Aldrich). In order to quantify metabolic activity, absorbance at 540 nm was measured in DMSO containing soluble formazan blue (Berthold technologies, Germany). DMSO was used as blank

Analysis of SGSC ploidity was performed according to Protocol S1.

### Immunofluorescence staining

SGSCs of 3 different donors in the passages 8, 14 and 21, each were cultured on 2- or 4- well chamber slides (BD Biosciences, Franklin Lakes, USA) until they reached confluence. Afterwards, samples were washed twice with PBS and fixed with 4% paraformaldehyde (PFA, Merck, Germany) for 10 min, rinsed 3 times with PBS. Next, SGSCs were incubated for 10 min with TritonX 0.1% (Fluka, Germany) containing 4',6-diamidin-2-phenylindol dihydrochlorid (DAPI, 1 µg/ml, Roche, Schweiz) and rinsed 3 times with PBS. Subsequently, samples were blocked in 10% goat serum (Vector Laboratories, CA, USA) for 20 min at room temperature. Primary antibodies against: Nestin (1:100, monoclonal, Millipore), Nestin (1:500, polyclonal, Abcam), Octamer binding transcription factor 4 (Oct4, 1:100, polyclonal, Santa Cruz), Sex determining region Y-box 2 (SOX2, 1:200, monoclonal, Cell Signaling), Neurofilament (NF H, 1:500, polyclonal, Serotec, NF M, 1:100, polyclonal, Santa Cruz, NF L, 1:200, polyclonal, Thermo Scientific), Neurofilament Mix L, M, H (1:200, monoclonal, Millipore), Keratin 14 (K14, 1:250, monoclonal, Santa Cruz), Keratin 19 (K19, 1:50, monoclonal, Sigma), Integrin alpha 6 (Iα6, 1:100, Santa Cruz), Mucin (1:500, monoclonal, Santa Cruz), Carcinoembryonic antigen (CEA, 1:500, polyclonal, Abcam), Alkaline phosphatase (AP, 1:50, monoclonal, R&D), Amylase (1:500, monoclonal, Santa Cruz), p63 (1:500, monoclonal, Santa Cruz), Vimentin (1:100, monoclonal, Dako) were incubated in a humid chamber for 1 h at 37°C or 4°C overnight in TBS-T (tris-buffered saline-triton X: 150 mM NaCl, 10 mM Tris (pH 8.8), 0.05% TritonX) containing 0.1% bovine serum albumin. Next, samples were washed 3 times with PBS and incubated with the secondary antibody: Cy3-labeled anti-mouse IgG (1:500), FITC-labelled anti-rabbit IgG (1:500) or Cy3-labeled anti-rat IgG (1:400) (all Jackson ImmunoResearch, USA) in a humid chamber for 1 h at 37°C. Finally, samples were washed 3 times in PBS, mounted in Vectashield mounting medium (Vector Laboratories, CA, USA) and analyzed by fluorescence microscopy (Observer, Zeiss, Germany). The negative controls were incubated with the corresponding IgG (Santa Cruz) instead of the primary antibody or just with the secondary antibody. Both methods resulted in a nonspecific, diffuse, faint staining (data not shown). 

### RNA isolation and quantitative PCR

RNA isolation and qPCR were carried out as previously published [75] for SGSCs of6 different donors. In addition 500 ng of total RNA was used and the following primers (all purchased from Qiagen): β-actin (146 bp), Nestin (75 bp), Nanog (90 bp), Oct4 (77 bp), SOX2 (64 bp), β3Tubulin (β3T; 78 bp), NF L (99 bp), K14 (76 bp), K19 (117 bp), Involucrin (120 bp), p63 (130 bp), Iα6 (142 bp), AP (110 bp), α smoth muscle actin (αSMA; 83 bp), von Willebrand factor (vWF, 108 bp), Albumin (106bp),Amylase (96bp), nerve growth factor receptor (NGFR) (118 bp), sex determining region Y-box 9 (SOX9) (111 bp) and platelet endothelial cell adhesion molecule (PECAM) (144 bp). Generated PCR products were in parts separated by capillary gel electrophoresis (QIAxcel; Qiagen).

### Time lapse microscopy

The time lapse microscope (Observer, Zeiss, Germany) is a microscope equipped with an incubator (37°C, 5% CO_2_) which takes consecutive pictures during the *in vitro* cell propagation. To observe outgrowth and proliferation of cells from the isolated sweat gland pictures were taken every 30 min from day 2 until day 10. 

### Cytokine profile

For the analysis of the secreted cytokines, 2.5*10^5^ cells were seeded in one well of a 6 well culture plate in triplets. After 24 h medium was changed and cells were cultured for further 24 h. Next, medium was collected and cytokine release was evaluated using a cytokine array or an angiogenesis array (both R&D Systems, Minneapolis, USA) following manufacturer’s instructions. Medium without cells was used as negative control. For detection we used the Western Lightning Plus-ECL (Perkin Elmer, USA). The emerging chemiluminescence was measured with the Fusion-SL (Vilber Lourmat, Germany). 

### Induction of mesodermal differentiation

For induction of adipogenic, chondrogenic and osteogenic differentiation SGSCs and BMSCs were treated with medium containing different supplements and growth factors for 21 days [61,76,77]. Briefly for adipogenic differentiation 3*10^3^ cells/cm^2^ were seeded and after 48 h adipogenesis was induced by differentiation medium (DMEM-10 supplemented with 200 µM indomethacin, 0.5 µM 3-isobutyl-1-methyl‑xanthine (IBMX), 1 µM dexamethasone and 10 µg/ml insulin (all Sigma-Aldrich, Germany). Every 3-4 days the used medium was changed between differentiation and maintenance medium (DMEM-10 with 10 µg/ml insulin). For detection of lipid vacuoles cells were stained with Oil Red O solution and counterstained with haematoxylin. 

For chondrogenic differentiation 5*10^5^ cells were pelleted by centrifugation at the bottom of a tube. After 24 h medium was changed to differentiation medium (DMEM supplemented with 1% 1x insulin-transferrin-selenite (ITS, BD Biosciences, USA), 1 mM sodium pyruvate, 40 µg/ml L-proline, 100 µg/ml L-ascorbic acid-phosphate, 100 nM dexamethasone and 10 ng/ml transforming growth factor-β3 (all Sigma-Aldrich, Germany)). Cryosections of 12 µm thickness were generated from micromass cultures frozen in Tissue Tek (Sakura, Netherlands) and subsequently stained with 1% alcian blue solution (Sigma-Aldrich, Germany) overnight. Additional detection of Collagen type II was done by immunhistochemistry staining. 

For osteogenic differentiation 3*10^3^ cells/cm^2^ were seeded, after 48 h osteogenesis was induced by the addition of differentiation medium (DMEM-10 supplemented with 10 mM β-glycerophosphate, 50 µM L-ascorbic acid-phosphate, 100 nM dexamethasone). Medium was changed every 3‑4 days. Alkaline phosphatase kit (Sigma Aldrich, Germany) was used according to the manufacturer’s protocol for the estimation of osteogenic differentiation. Corresponding controls were cultivated in medium without soluble factors or maintenance medium for 21 days.

### Statistical analysis

GraphPad Prism 5 software (GraphPad Software, CA, USA) was used for statistic analyses. The 2way ANOVA (analysis of variance) was used to calculate the influences of the cultivation and passage number on the relative gene expression level/β-actin of SGSCs. Differences among means were considered significant when p<0.05. Bonferroni post hoc test was used to determine significant differences between genes . 

## Supporting Information

Figure S1 **Controls of the induced differentiation of SGSCs and BMSCs were cultivated in medium without soluble factors or maintenance medium for 21 days.** Except of a slight alcian blue staining at the edges of the very spongy MMBs of SGSCs and BMSCs, no positive cells could be detected within the corresponding negative controls. Oil red O, Collagen Type II, AP Nuclei were stained with haematoxilin. Scale bars 100 μm Alcian blue Scale bars 200 μm .
(TIF)

Figure S2 **DNA amount determination for exclusion of aneuploidy via 7-AAD.** SGSCs were analyzed in passage 9 (light gray), passage 14 (dark gray) and passage 20 (black). Beside usual peaks for diploid cells (first peak) and tetraploid cells (mitotic cells, second peak) no other peak and thus no changes in ploidity could be observed within analyzed SGSCs during propagation.
(TIF)

Figure S3 **Protein expression profile of different cell populations.** A) The key marker Nestin (red) distinguishes SGSCs not only from BMSCs but also from dermal fibroblasts, which expressed Nestin in 5% respectively 1% of the cells. B) SGSCs were also positive for Vimentin (red). Nuclei were stained with DAPI. Scale bars 100 μm.
(TIF)

Figure S4 **Analysis of potential origins of SGSCs via qPCR.** Markers for cell types like neural crest cells (NGFR, SOX9) and endothelial cells (PECAM) could be detected within *in* *situ* sweat gland preparation, passage 0 and passage 14.
(TIF)

Protocol S1 **Ploidity was characterized via 7-AAD staining and subsequent FACS analysis.**
(DOCX)

## References

[1] Dahlstrand J, Zimmerman LB, McKay RD, Lendahl U (1992) Characterization of the human nestin gene reveals a close evolutionary relationship to neurofilaments. J Cell Sci 103 ( 2): 589-597. PubMed: 1478958. 1478958 10.1242/jcs.103.2.589

[2] Yaworsky PJ, Kappen C (1999) Heterogeneity of neural progenitor cells revealed by enhancers in the nestin gene. Dev Biol 205: 309-321. doi:10.1006/dbio.1998.9035. PubMed: 9917366. 9917366 PMC3938161

[3] Lendahl U, Zimmerman LB, McKay RD (1990) CNS stem cells express a new class of intermediate filament protein. Cell 60: 585-595. doi:10.1016/0092-8674(90)90662-X. PubMed: 1689217. 1689217

[4] Lothian C, Lendahl U (1997) An evolutionarily conserved region in the second intron of the human nestin gene directs gene expression to CNS progenitor cells and to early neural crest cells. Eur J Neurosci 9: 452-462. doi:10.1111/j.1460-9568.1997.tb01622.x. PubMed: 9104587. 9104587

[5] Wiese C, Rolletschek A, Kania G, Blyszczuk P, Tarasov KV et al. (2004) Nestin expression--a property of multi-lineage progenitor cells? Cell Mol Life Sci 61: 2510-2522. doi:10.1007/s00018-004-4144-6. PubMed: 15526158. 15526158 PMC11924557

[6] Amoh Y, Li L, Katsuoka K, Hoffman RM (2009) Multipotent nestin-expressing hair follicle stem cells. J Dermatol 36: 1-9. doi:10.1111/j.1346-8138.2008.00578.x. PubMed: 19207430. 19207430

[7] Hoffman RM (2007) The potential of nestin-expressing hair follicle stem cells in regenerative medicine. Expert Opin Biol Ther 7: 289-291. doi:10.1517/14712598.7.3.289. PubMed: 17309321. 17309321

[8] Li L, Mignone J, Yang M, Matic M, Penman S et al. (2003) Nestin expression in hair follicle sheath progenitor cells. Proc Natl Acad Sci U S A 100: 9958-9961. doi:10.1073/pnas.1733025100. PubMed: 12904579. 12904579 PMC187900

[9] Toma JG, Akhavan M, Fernandes KJ, Barnabé-Heider F, Sadikot A et al. (2001) Isolation of multipotent adult stem cells from the dermis of mammalian skin. Nat Cell Biol 3: 778-784. doi:10.1038/ncb0901-778. PubMed: 11533656. 11533656

[10] Park D, Xiang AP, Mao FF, Zhang L, Di CG et al. (2010) Nestin is required for the proper self-renewal of neural stem cells. Stem Cells 28: 2162-2171. doi:10.1002/stem.541. PubMed: 20963821. 20963821

[11] Sahlgren CM, Mikhailov A, Hellman J, Chou YH, Lendahl U et al. (2001) Mitotic reorganization of the intermediate filament protein nestin involves phosphorylation by cdc2 kinase. J Biol Chem 276: 16456-16463. doi:10.1074/jbc.M009669200. PubMed: 11278541. 11278541

[12] Kruse C, Bodó E, Petschnik AE, Danner S, Tiede S et al. (2006) Towards the development of a pragmatic technique for isolating and differentiating nestin-positive cells from human scalp skin into neuronal and glial cell populations: generating neurons from human skin? Exp Dermatol 15: 794-800. doi:10.1111/j.1600-0625.2006.00471.x. PubMed: 16984261. 16984261

[13] Toma JG, McKenzie IA, Bagli D, Miller FD (2005) Isolation and characterization of multipotent skin-derived precursors from human skin. Stem Cells 23: 727-737. doi:10.1634/stemcells.2004-0134. PubMed: 15917469. 15917469

[14] Kruse C, Birth M, Rohwedel J, Assmuth [!(surname)!], Goepel A et al. (2004) Pluripotency of adult stem cells derived from human and rat pancreas. Appl Phys A 79: 1617-1624.

[15] Gorjup E, Danner S, Rotter N, Habermann J, Brassat U et al. (2009) Glandular tissue from human pancreas and salivary gland yields similar stem cell populations. Eur J Cell Biol 88: 409-421. doi:10.1016/j.ejcb.2009.02.187. PubMed: 19410331. 19410331

[16] Petschnik AE, Klatte JE, Evers LH, Kruse C, Paus R et al. (2010) Phenotypic indications that human sweat glands are a rich source of nestin-positive stem cell populations. Br J Dermatol 162: 380-383. doi:10.1111/j.1365-2133.2009.09512.x. PubMed: 19772523. 19772523

[17] Tiede S, Kloepper JE, Ernst N, Poeggeler B, Kruse C et al. (2009) Nestin in human skin: exclusive expression in intramesenchymal skin compartments and regulation by leptin. J Invest Dermatol 129: 2711-2720. doi:10.1038/jid.2009.148. PubMed: 19554024. 19554024

[18] Amoh Y, Kanoh M, Niiyama S, Hamada Y, Kawahara K et al. (2009) Human hair follicle pluripotent stem (hfPS) cells promote regeneration of peripheral-nerve injury: an advantageous alternative to ES and iPS cells. J Cell Biochem 107: 1016-1020. doi:10.1002/jcb.22204. PubMed: 19507228. 19507228

[19] Amoh Y, Hoffman RM (2010) Isolation and culture of hair follicle pluripotent stem (hfPS) cells and their use for nerve and spinal cord regeneration. Methods Mol Biol 585: 401-420. doi:10.1007/978-1-60761-380-0_28. PubMed: 19908019. 19908019

[20] Aki R, Amoh Y, Li L, Katsuoka K, Hoffman RM (2010) Nestin-expressing interfollicular blood vessel network contributes to skin transplant survival and wound healing. J Cell Biochem 110: 80-86. PubMed: 20225276. 20225276 10.1002/jcb.22512

[21] Amoh Y, Li L, Yang M, Moossa AR, Katsuoka K et al. (2004) Nascent blood vessels in the skin arise from nestin-expressing hair-follicle cells. Proc Natl Acad Sci U S A 101: 13291-13295. doi:10.1073/pnas.0405250101. PubMed: 15331785. 15331785 PMC516562

[22] Danner S, Kremer M, Petschnik AE, Nagel S, Zhang Z et al. (2012) The Use of Human Sweat Gland-Derived Stem Cells for Enhancing Vascularization during Dermal Regeneration. J Invest Dermatol 132: 1707-1716. doi:10.1038/jid.2012.31. PubMed: 22377762. 22377762

[23] Biedermann T, Pontiggia L, Böttcher-Haberzeth S, Tharakan S, Braziulis E et al. (2010) Human eccrine sweat gland cells can reconstitute a stratified epidermis. J Invest Dermatol 130: 1996-2009. doi:10.1038/jid.2010.83. PubMed: 20376062. 20376062

[24] Rittié L, Sachs DL, Orringer JS, Voorhees JJ, Fisher GJ (2012) Eccrine Sweat Glands are Major Contributors to Reepithelialization of Human Wounds. Am J Pathol 182: 163-171. PubMed: 23159944. 23159944 10.1016/j.ajpath.2012.09.019 PMC3538027

[25] Lu CP, Polak L, Rocha AS, Pasolli HA, Chen SC et al. (2012) Identification of stem cell populations in sweat glands and ducts reveals roles in homeostasis and wound repair. Cell 150: 136-150. doi:10.1016/j.cell.2012.04.045. PubMed: 22770217. 22770217 PMC3423199

[26] Ji KH, Xiong J, Hu KM, Fan LX, Liu HQ (2008) Simultaneous expression of Oct4 and genes of three germ layers in single cell-derived multipotent adult progenitor cells. Ann Hematol 87: 431-438. doi:10.1007/s00277-008-0470-3. PubMed: 18338169. 18338169 PMC2324127

[27] Chambers I, Colby D, Robertson M, Nichols J, Lee S et al. (2003) Functional expression cloning of Nanog, a pluripotency sustaining factor in embryonic stem cells. Cell 113: 643-655. doi:10.1016/S0092-8674(03)00392-1. PubMed: 12787505. 12787505

[28] Ernst N, Tiede S, Tronnier V, Kruse C, Zechel C et al. (2010) An improved, standardised protocol for the isolation, enrichment and targeted neural differentiation of Nestin+ progenitors from adult human dermis. Exp Dermatol 19: 549-555. doi:10.1111/j.1600-0625.2009.01041.x. PubMed: 20100195. 20100195

[29] Ersch J, Stallmach T (1999) Assessing gestational age from histology of fetal skin: an autopsy study of 379 fetuses. Obstet Gynecol 94: 753-757. doi:10.1016/S0029-7844(99)00379-8. PubMed: 10546723. 10546723

[30] Holbrook KA, Minami SI (1991) Hair follicle embryogenesis in the human. Characterization of events in vivo and in vitro. Ann N Y Acad Sci 642: 167-196. PubMed: 1809080. 1809080

[31] Moll I, Moll R (1992) Changes of expression of intermediate filament proteins during ontogenesis of eccrine sweat glands. J Invest Dermatol 98: 777-785. doi:10.1111/1523-1747.ep12499950. PubMed: 1569327. 1569327

[32] Cowin P, Wysolmerski J (2010) Molecular mechanisms guiding embryonic mammary gland development. Cold Spring Harb Perspect Biol 2: a003251. doi:10.1101/cshperspect.a003251. PubMed: 20484386. 20484386 PMC2869520

[33] Li H, Cherukuri P, Li N, Cowling V, Spinella M et al. (2007) Nestin is expressed in the basal/myoepithelial layer of the mammary gland and is a selective marker of basal epithelial breast tumors. Cancer Res 67: 501-510. doi:10.1158/0008-5472.CAN-05-4571. PubMed: 17234757. 17234757

[34] Kolar Z, Ehrmann J Jr., Turashvili G, Bouchal J, Mokry J (2007) A novel myoepithelial/progenitor cell marker in the breast? Virchows Arch 450: 607-609. doi:10.1007/s00428-007-0403-x. PubMed: 17429688. 17429688

[35] Rapoport DH, Schicktanz S, Gürleyik E, Zühlke C, Kruse C (2009) Isolation and in vitro cultivation turns cells from exocrine human pancreas into multipotent stem-cells. Ann Anat 191: 446-458. doi:10.1016/j.aanat.2009.07.002. PubMed: 19716277. 19716277

[36] Fong H, Hohenstein KA, Donovan PJ (2008) Regulation of self-renewal and pluripotency by Sox2 in human embryonic stem cells. Stem Cells 26: 1931-1938. doi:10.1634/stemcells.2007-1002. PubMed: 18388306. 18388306

[37] Reubinoff BE, Pera MF, Fong CY, Trounson A, Bongso A (2000) Embryonic stem cell lines from human blastocysts: somatic differentiation in vitro. Nat Biotechnol 18: 399-404. doi:10.1038/74447. PubMed: 10748519. 10748519

[38] Schöler HR, Ruppert S, Suzuki N, Chowdhury K, Gruss P (1990) New type of POU domain in germ line-specific protein Oct-4. Nature 344: 435-439. doi:10.1038/344435a0. PubMed: 1690859. 1690859

[39] Tai MH, Chang CC, Kiupel M, Webster JD, Olson LK et al. (2005) Oct4 expression in adult human stem cells: evidence in support of the stem cell theory of carcinogenesis. Carcinogenesis 26: 495-502. PubMed: 15513931. 15513931 10.1093/carcin/bgh321

[40] Yu H, Fang D, Kumar SM, Li L, Nguyen TK et al. (2006) Isolation of a novel population of multipotent adult stem cells from human hair follicles. Am J Pathol 168: 1879-1888. doi:10.2353/ajpath.2006.051170. PubMed: 16723703. 16723703 PMC1606635

[41] Yang C, Zhu P, Yan L, Chen L, Meng R et al. (2009) Dynamic changes in matrix metalloproteinase 9 and tissue inhibitor of metalloproteinase 1 levels during wound healing in diabetic rats. J Am Podiatr Med Assoc 99: 489-496. PubMed: 19917734. 19917734 10.7547/0990489

[42] Distler JH, Hirth A, Kurowska-Stolarska M, Gay RE, Gay S et al. (2003) Angiogenic and angiostatic factors in the molecular control of angiogenesis. Q J Nucl Med 47: 149-161. PubMed: 12897707. 12897707

[43] Werner S, Grose R (2003) Regulation of wound healing by growth factors and cytokines. Physiol Rev 83: 835-870. PubMed: 12843410. 12843410 10.1152/physrev.2003.83.3.835

[44] Barrientos S, Stojadinovic O, Golinko MS, Brem H, Tomic-Canic M (2008) Growth factors and cytokines in wound healing. Wound Repair Regen 16: 585-601. doi:10.1111/j.1524-475X.2008.00410.x. PubMed: 19128254. 19128254

[45] Gurtner GC, Werner S, Barrandon Y, Longaker MT (2008) Wound repair and regeneration. Nature 453: 314-321. doi:10.1038/nature07039. PubMed: 18480812. 18480812

[46] Sinha S, Fuchs E (2001) Identification and dissection of an enhancer controlling epithelial gene expression in skin. Proc Natl Acad Sci U_S_A 98: 2455-2460. doi:10.1073/pnas.051633598. PubMed: 11226260. 11226260 PMC30159

[47] Gho CG, Braun JE, Tilli CM, Neumann HA, Ramaekers FC (2004) Human follicular stem cells: their presence in plucked hair and follicular cell culture. Br J Dermatol 150: 860-868. doi:10.1111/j.1365-2133.2004.05862.x. PubMed: 15149497. 15149497

[48] Purkis PE, Steel JB, Mackenzie IC, Nathrath WB, Leigh IM et al. (1990) Antibody markers of basal cells in complex epithelia. J Cell Sci 97 ( 1): 39-50. PubMed: 1701769. 1701769 10.1242/jcs.97.1.39

[49] Pontiggia L, Biedermann T, Meuli M, Widmer D, Böttcher-Haberzeth S et al. (2009) Markers to evaluate the quality and self-renewing potential of engineered human skin substitutes in vitro and after transplantation. J Invest Dermatol 129: 480-490. doi:10.1038/jid.2008.254. PubMed: 18719609. 18719609

[50] Cotsarelis G (2006) Epithelial stem cells: a folliculocentric view. J Invest Dermatol 126: 1459-1468. doi:10.1038/sj.jid.5700376. PubMed: 16778814. 16778814

[51] Akiyama M, Smith LT, Shimizu H (2000) Changing patterns of localization of putative stem cells in developing human hair follicles. J Invest Dermatol 114: 321-327. doi:10.1046/j.1523-1747.2000.00857.x. PubMed: 10651993. 10651993

[52] Larouche D, Hayward C, Cuffley K, Germain L (2005) Keratin 19 as a stem cell marker in vivo and in vitro. Methods Mol Biol 289: 103-110. PubMed: 15502175. 15502175 10.1385/1-59259-830-7:103

[53] Kloepper JE, Tiede S, Brinckmann J, Reinhardt DP, Meyer W et al. (2008) Immunophenotyping of the human bulge region: the quest to define useful in situ markers for human epithelial hair follicle stem cells and their niche. Exp Dermatol 17: 592-609. doi:10.1111/j.1600-0625.2008.00720.x. PubMed: 18558994. 18558994

[54] Bouvard C, Gafsou B, Dizier B, Galy-Fauroux I, Lokajczyk A et al. (2010) alpha6-integrin subunit plays a major role in the proangiogenic properties of endothelial progenitor cells. Arterioscler Thromb Vasc Biol 30: 1569-1575. doi:10.1161/ATVBAHA.110.209163. PubMed: 20508204. 20508204

[55] Chung J, Bachelder RE, Lipscomb EA, Shaw LM, Mercurio AM (2002) Integrin (alpha 6 beta 4) regulation of eIF-4E activity and VEGF translation: a survival mechanism for carcinoma cells. J Cell Biol 158: 165-174. doi:10.1083/jcb.200112015. PubMed: 12105188. 12105188 PMC2173018

[56] Spradling A, Drummond-Barbosa D, Kai T (2001) Stem cells find their niche. Nature 414: 98-104. doi:10.1038/35102160. PubMed: 11689954. 11689954

[57] Yu KR, Yang SR, Jung JW, Kim H, Ko K et al. (2012) CD49f enhances multipotency and maintains stemness through the direct regulation of OCT4 and SOX2. Stem Cells 30: 876-887. doi:10.1002/stem.1052. PubMed: 22311737. 22311737

[58] Carroll DK, Carroll JS, Leong CO, Cheng F, Brown M et al. (2006) p63 regulates an adhesion programme and cell survival in epithelial cells. Nat Cell Biol 8: 551-561. doi:10.1038/ncb1420. PubMed: 16715076. 16715076

[59] Schreder A, Pierard GE, Paquet P, Reginster MA, Pierard-Franchimont C et al. (2010) Facing towards epidermal stem cells (Review ). Int J Mol Med 26: 171-174 PubMed: 20596595. 10.3892/ijmm_00000449 20596595

[60] Senoo M, Pinto F, Crum CP, McKeon F (2007) p63 Is essential for the proliferative potential of stem cells in stratified epithelia. Cell 129: 523-536. doi:10.1016/j.cell.2007.02.045. PubMed: 17482546. 17482546

[61] Pittenger MF, Mackay AM, Beck SC, Jaiswal RK, Douglas R et al. (1999) Multilineage potential of adult human mesenchymal stem cells. Science 284: 143-147. doi:10.1126/science.284.5411.143. PubMed: 10102814. 10102814

[62] Jiang Y, Jahagirdar BN, Reinhardt RL, Schwartz RE, Keene CD et al. (2002) Pluripotency of mesenchymal stem cells derived from adult marrow. Nature 418: 41-49. doi:10.1038/nature00870. PubMed: 12077603. 12077603

[63] Eliasson C, Sahlgren C, Berthold CH, Stakeberg J, Celis JE et al. (1999) Intermediate filament protein partnership in astrocytes. J Biol Chem 274: 23996-24006. doi:10.1074/jbc.274.34.23996. PubMed: 10446168. 10446168

[64] Mokrý J, Ehrmann J, Karbanová J, Cízková D, Soukup T et al. (2008) Expression of intermediate filament nestin in blood vessels of neural and non-neural tissues. Acta Med (Hradec Kralove) 51: 173-179. PubMed: 19271685. 10.14712/18059694.2017.20 19271685

[65] Schmidt A, Ladage D, Schinköthe T, Klausmann U, Ulrichs C et al. (2006) Basic fibroblast growth factor controls migration in human mesenchymal stem cells. Stem Cells 24: 1750-1758. doi:10.1634/stemcells.2005-0191. PubMed: 16822883. 16822883

[66] Steinert PM, Chou YH, Prahlad V, Parry DA, Marekov LN et al. (1999) A high molecular weight intermediate filament-associated protein in BHK-21 cells is nestin, a type VI intermediate filament protein. Limited co-assembly in vitro to form heteropolymers with type III vimentin and type IV alpha-internexin. J Biol Chem 274: 9881-9890. doi:10.1074/jbc.274.14.9881. PubMed: 10092680. 10092680

[67] Chou YH, Khuon S, Herrmann H, Goldman RD (2003) Nestin promotes the phosphorylation-dependent disassembly of vimentin intermediate filaments during mitosis. Mol Cell Biol 14: 1468-1478. doi:10.1091/mbc.E02-08-0545. PubMed: 12686602. PMC153115 12686602

[68] Chang L, Goldman RD (2004) Intermediate filaments mediate cytoskeletal crosstalk. Nat Rev Mol Cell Biol 5: 601-613. doi:10.1038/nrm1438. PubMed: 15366704. 15366704

[69] Fernandes KJ, McKenzie IA, Mill P, Smith KM, Akhavan M et al. (2004) A dermal niche for multipotent adult skin-derived precursor cells. Nat Cell Biol 6: 1082-1093. doi:10.1038/ncb1181. PubMed: 15517002. 15517002

[70] Sieber-Blum M, Grim M (2004) The adult hair follicle: cradle for pluripotent neural crest stem cells. Birth Defects Res C Embryo TODAY 72: 162-172. doi:10.1002/bdrc.20008. PubMed: 15269890. 15269890

[71] Wong CE, Paratore C, Dours-Zimmermann MT, Rochat A, Pietri T et al. (2006) Neural crest-derived cells with stem cell features can be traced back to multiple lineages in the adult skin. J Cell Biol 175: 1005-1015. doi:10.1083/jcb.200606062. PubMed: 17158956. 17158956 PMC2064709

[72] Aihara M, Sugawara K, Torii S, Hosaka M, Kurihara H et al. (2004) Angiogenic endothelium-specific nestin expression is enhanced by the first intron of the nestin gene. Lab Invest 84: 1581-1592. doi:10.1038/labinvest.3700186. PubMed: 15502861. 15502861

[73] Mokrý J, Cízková D, Filip S, Ehrmann J, Osterreicher J et al. (2004) Nestin expression by newly formed human blood vessels. Stem Cells Dev 13: 658-664. doi:10.1089/scd.2004.13.658. PubMed: 15684833. 15684833

[74] Kim DS, Cho HJ, Choi HR, Kwon SB, Park KC (2004) Isolation of human epidermal stem cells by adherence and the reconstruction of skin equivalents. Cell Mol Life Sci 61: 2774-2781. doi:10.1007/s00018-004-4288-4. PubMed: 15549181. 15549181 PMC11924481

[75] Petschnik AE, Fell B, Tiede S, Habermann JK, Pries R et al. (2011) A novel xenogeneic co-culture system to examine neuronal differentiation capability of various adult human stem cells. PLOS ONE 6: e24944. doi:10.1371/journal.pone.0024944. PubMed: 21935488. 21935488 PMC3173484

[76] Jaiswal N, Haynesworth SE, Caplan AI, Bruder SP (1997) Osteogenic differentiation of purified, culture-expanded human mesenchymal stem cells in vitro. J Cell Biochem 64: 295-312. doi:10.1002/(SICI)1097-4644(199702)64:2. PubMed: 9027589. 9027589

[77] Mackay AM, Beck SC, Murphy JM, Barry FP, Chichester CO et al. (1998) Chondrogenic differentiation of cultured human mesenchymal stem cells from marrow. Tissue Eng 4: 415-428. doi:10.1089/ten.1998.4.415. PubMed: 9916173. 9916173

