# Supplementary figures and images for: Multipotent Nestin-Positive Stem Cells Reside in the Stroma of Human Eccrine and Apocrine Sweat Glands and Can Be Propagated Robustly In Vitro
Source: PLoS One. 2013 Oct 24;8(10):e78365. doi: 10.1371/journal.pone.0078365 (PMC3813437; doi:10.1371/journal.pone.0078365)

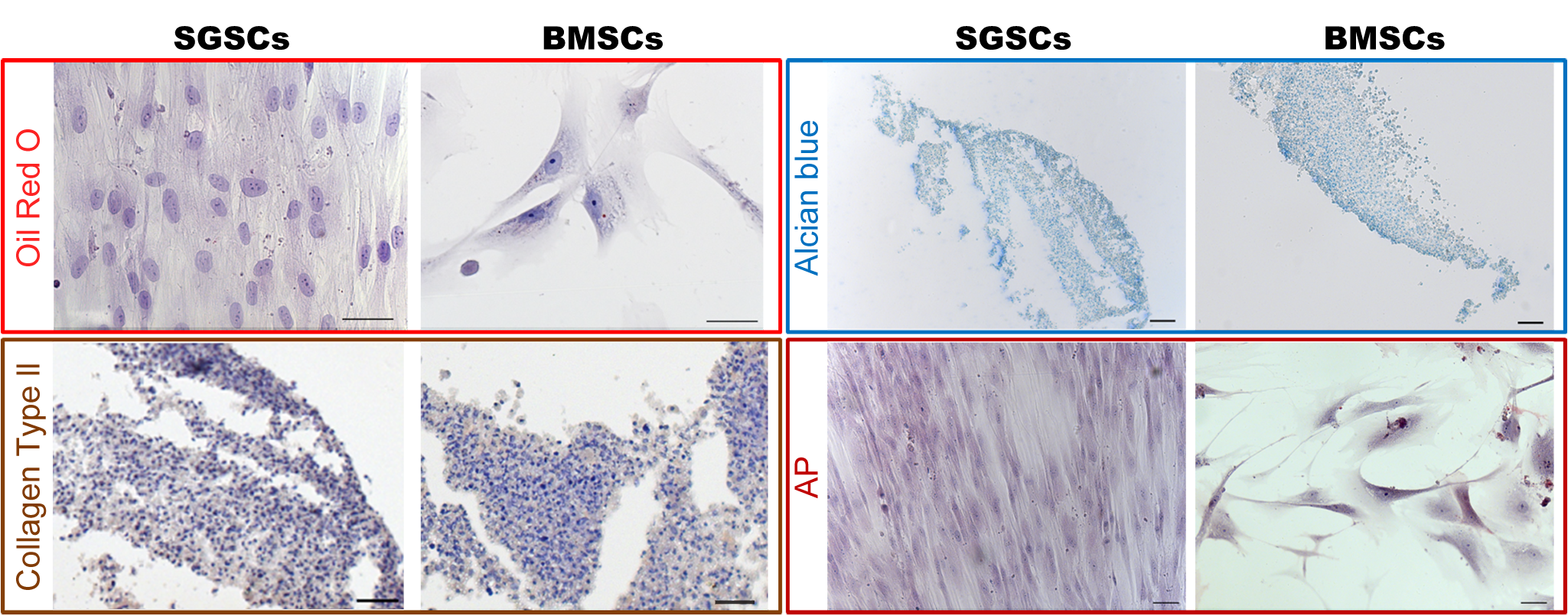

Supplement: Figure S1 — Controls of the induced differentiation of SGSCs and BMSCs were cultivated in medium without soluble factors or maintenance medium for 21 days. Except of a slight alcian blue staining at the edges of the very spongy MMBs of SGSCs and BMSCs, no positive cells could be detected within the corresponding negative controls. Oil red O, Collagen Type II, AP Nuclei were stained with haematoxilin. Scale bars 100 μm Alcian blue Scale bars 200 μm . (TIF) [file pone.0078365.s001.tif]

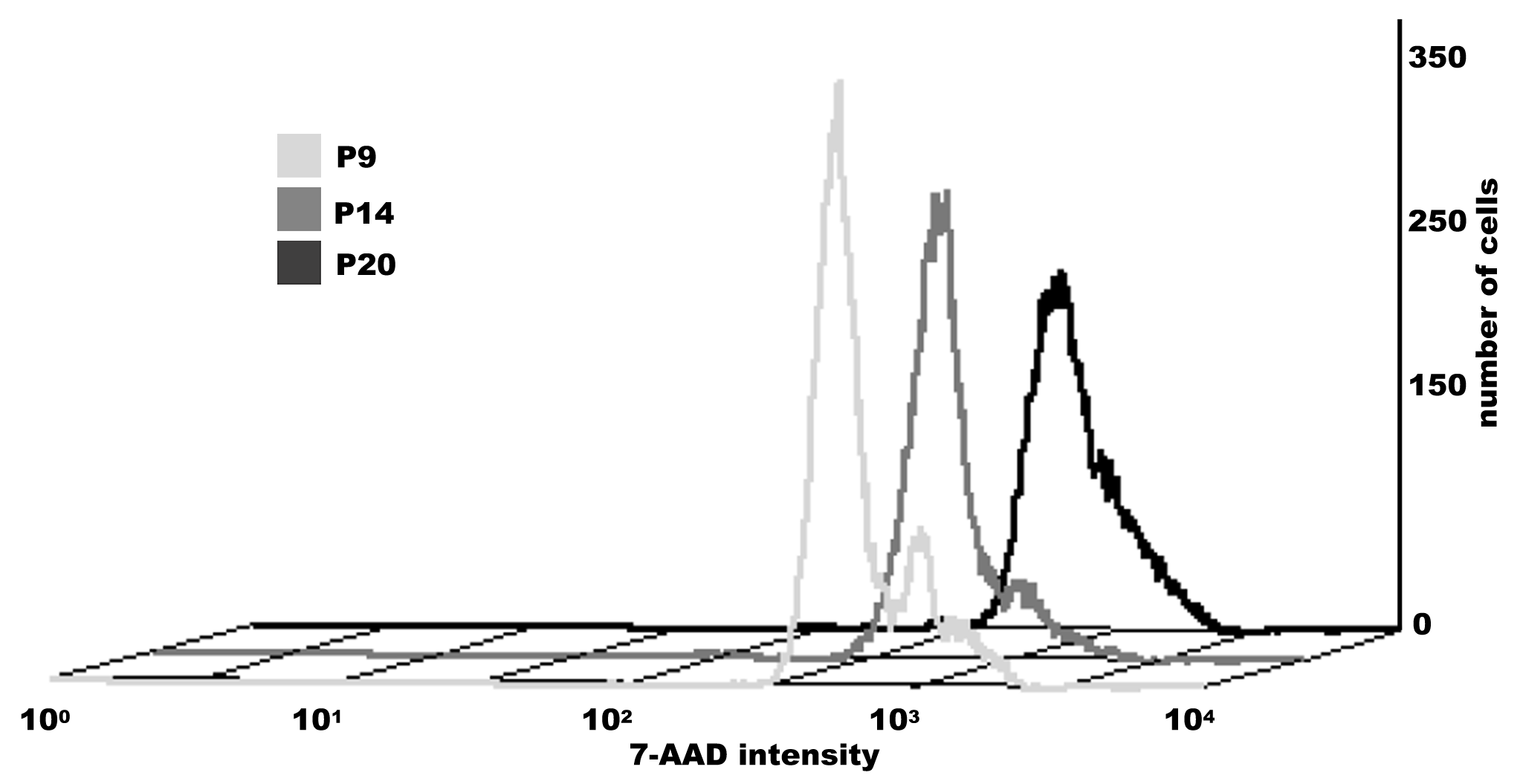

Supplement: Figure S2 — DNA amount determination for exclusion of aneuploidy via 7-AAD. SGSCs were analyzed in passage 9 (light gray), passage 14 (dark gray) and passage 20 (black). Beside usual peaks for diploid cells (first peak) and tetraploid cells (mitotic cells, second peak) no other peak and thus no changes in ploidity could be observed within analyzed SGSCs during propagation. (TIF) [file pone.0078365.s002.tif]

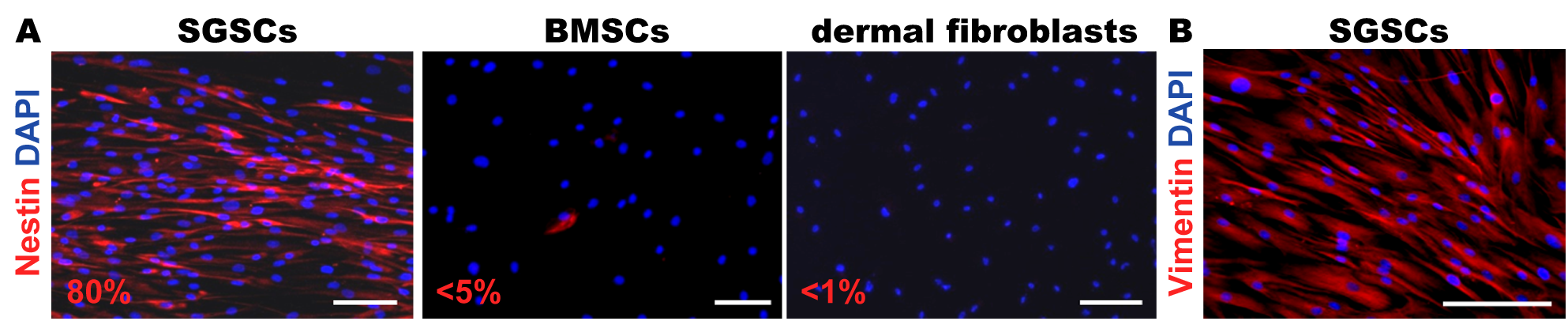

Supplement: Figure S3 — Protein expression profile of different cell populations. A) The key marker Nestin (red) distinguishes SGSCs not only from BMSCs but also from dermal fibroblasts, which expressed Nestin in 5% respectively 1% of the cells. B) SGSCs were also positive for Vimentin (red). Nuclei were stained with DAPI. Scale bars 100 μm. (TIF) [file pone.0078365.s003.tif]

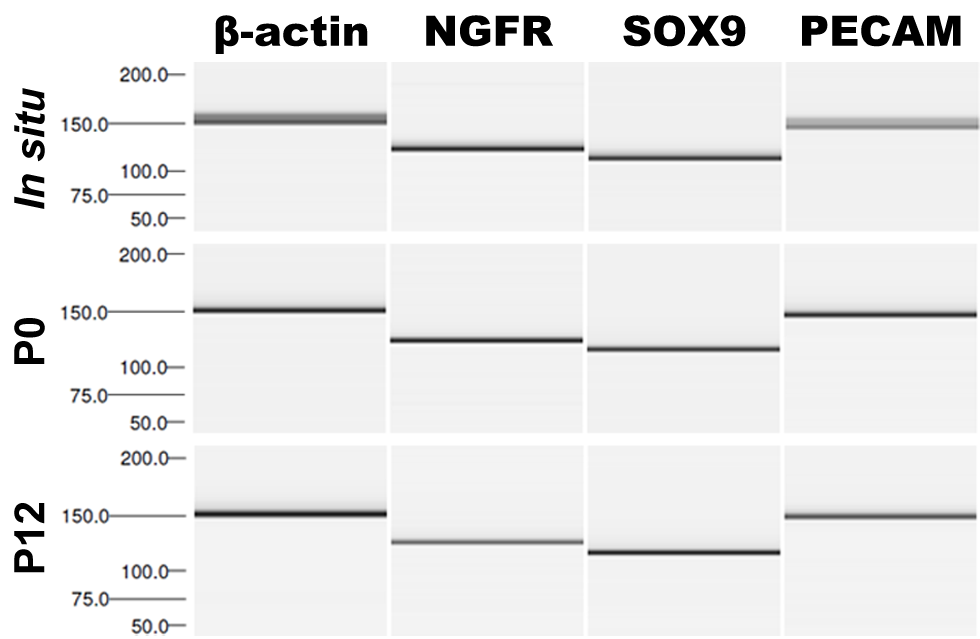

Supplement: Figure S4 — Analysis of potential origins of SGSCs via qPCR. Markers for cell types like neural crest cells (NGFR, SOX9) and endothelial cells (PECAM) could be detected within in situ sweat gland preparation, passage 0 and passage 14. (TIF) [file pone.0078365.s004.tif]
