# Supplementary material for: Multipotent Nestin-Positive Stem Cells Reside in the Stroma of Human Eccrine and Apocrine Sweat Glands and Can Be Propagated Robustly In Vitro
Source: PLoS One. 2013 Oct 24;8(10):e78365. doi: 10.1371/journal.pone.0078365 (PMC3813437; doi:10.1371/journal.pone.0078365)
Supplement: Protocol S1 — Ploidity was characterized via 7-AAD staining and subsequent FACS analysis. (DOCX) [file pone.0078365.s005.docx]

Protocol S5: SGSCs were harvested and fixed in 70% ethanol overnight at -20°C. Next day, DNA staining was performed using 7-aminoactinomycin D (7-AAD) viability staining solution (BioLegend, USA) for 10 min in dark, prior to flow cytometric analysis. Analysis was performed on a FACS calibur flow cytometer (Becton Dickinson, USA). For each sample, 20.000 events were collected and aggregated cells were gated out. The cell cycle analysis was performed using CellQuest Pro software.
